# The Chemistry of Cu_3_N and Cu_3_PdN Nanocrystals**

**DOI:** 10.1002/anie.202207013

**Published:** 2022-06-15

**Authors:** Mahsa Parvizian, Alejandra Duràn Balsa, Rohan Pokratath, Curran Kalha, Seungho Lee, Dietger Van den Eynden, Maria Ibáñez, Anna Regoutz, Jonathan De Roo

**Affiliations:** ^1^ Department of Chemistry University of Basel 4058 Basel Switzerland; ^2^ Department of Chemistry University College London 20 Gordon Street London WC1H 0AJ UK; ^3^ IST Austria Am Campus 1 3400 Klosterneuburg Austria

**Keywords:** Copper Nitride, Ligands, Nitrides, Reaction Mechanisms, Structural Analysis, Surface Chemistry

## Abstract

The precursor conversion chemistry and surface chemistry of Cu_3_N and Cu_3_PdN nanocrystals are unknown or contested. Here, we first obtain phase‐pure, colloidally stable nanocubes. Second, we elucidate the pathway by which copper(II) nitrate and oleylamine form Cu_3_N. We find that oleylamine is both a reductant and a nitrogen source. Oleylamine is oxidized by nitrate to a primary aldimine, which reacts further with excess oleylamine to a secondary aldimine, eliminating ammonia. Ammonia reacts with Cu^I^ to form Cu_3_N. Third, we investigated the surface chemistry and find a mixed ligand shell of aliphatic amines and carboxylates (formed in situ). While the carboxylates appear tightly bound, the amines are easily desorbed from the surface. Finally, we show that doping with palladium decreases the band gap and the material becomes semi‐metallic. These results bring insight into the chemistry of metal nitrides and might help the development of other metal nitride nanocrystals.

## Graphical abstract

Phase‐pure, colloidally stable Cu_3_N and Cu_3_PdN were obtained. The precursor conversion mechanism is elucidated; oleylamine is oxidized to a primary aldimine and the latter reacts further with oleylamine to a secondary aldimine, eliminating ammonia. Ammonia reacts with Cu^I^ to form Cu_3_N.

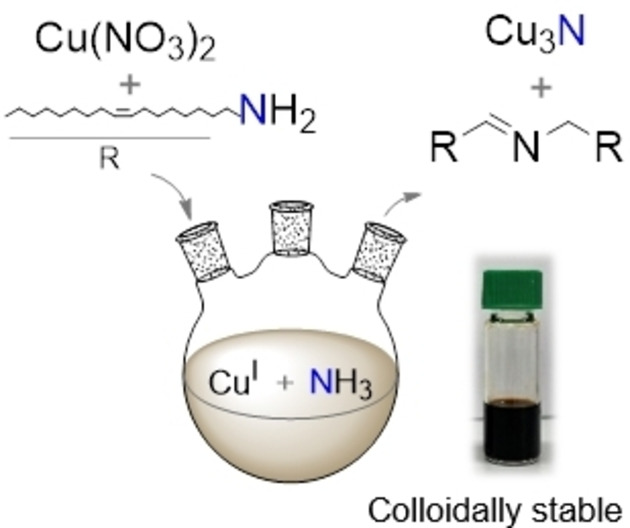

## Introduction

Metal nitrides are a versatile class of materials with increasing interest.^[1]^ Copper nitride (Cu_3_N) specifically has garnered attention as an inexpensive, non‐toxic material with potential applications in solar cells,^[2]^ high‐density optical data storage,^[3]^ and electrocatalysis (oxygen evolution and CO_2_ reduction).^[4]^ Cu_3_N is a semiconductor with a calculated indirect band gap of 1 eV and an anti‐ReO_3_ cubic crystal structure.[^2^, ^5^] The body center position can be occupied by dopants (e.g., palladium) forming structures such as Cu_3_Pd_*x*_N. Upon doping, the lattice constant increases,^[6]^ and the electronic structure of the material is reported to change from semiconducting to semi‐metallic.^[7]^

**Scheme 1:**
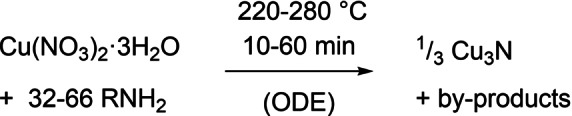
The range of conditions reported for the synthesis of colloidal Cu_3_N nanocrystals.

Bulk Cu_3_N forms at relatively low temperature but decomposes at higher temperature (475 °C) to metallic copper (under inert atmosphere) or copper oxide (in air).[^3^, ^8^] The first wet‐chemical synthesis of bulk Cu_3_N powders were based on aminolysis or the solvothermal decomposition of copper azides.[^8^, ^9^] Ultra‐small (2–4 nm) and colloidally stable nanocrystals of Cu_3_N have been synthesized from Cu(OMe)_2_ in benzylamine_,_ ^[10]^ or by aminolysis of Cu^I^ in pyridine.^[11]^ Larger colloidal Cu_3_N nanocubes have been obtained from copper nitrate and alkylamine (Scheme 1).[^4a^, ^4c^, ^12^] In most cases, additional solvent is added (usually 1‐octadecene, ODE). The equivalents of ligand (32–66), the reaction temperature (220–280 °C), and time (10–60 min) vary, as does the nature of the alkyl chain of the amine (oleylamine, hexadecylamine or octadecylamine). Changing the nature of the amine allows to tune the final size from 10 to 25 nm.^[12a]^

Unfortunately, the reaction mechanism and the by‐products are not well established. Some authors claim that nitrate is the nitrogen source, and thus nitrogen is supposed to be reduced from its highest oxidation state in nitrate to its lowest oxidation state in nitride, and this process is presumably catalyzed by the amine ligand.^[12c]^ Such an eight‐electron redox process is unlikely to happen in a single reaction. Other authors assume that Cu^II^ is first fully reduced to Cu^0^ and subsequently reacts with dinitrogen (a possible decomposition product of the nitrate complex).^[13]^ However, the nitrogen‐nitrogen bond in dinitrogen is extremely strong (946 kJ mol^−1^),^[14]^ and N_2_ is thus not likely to react with elemental copper at 250 °C. There is currently insufficient evidence to claim a mechanism for the precursor conversion. Regarding the crystallization mechanism, it was shown that first small, amorphous particles form, which subsequently ripen into Cu_3_N nanocubes.^[4c]^

The surface chemistry of Cu_3_N nanocrystals is also unknown. Although alkylamines are the only ligand present during the synthesis, there are several examples where the ligands can convert to other ligands during nanocrystal synthesis, leading to surprising surface chemistries.^[15]^ Given the importance of surface chemistry for nanocrystal applications it is imperative to elucidate and control it.^[16]^ Amines typically bind to the nanocrystal surface as Lewis basic (L‐type) ligands and feature an adsorption–desorption equilibrium that is often highly dynamic.^[17]^ However, in the case of copper‐based nanocrystals, amines have been observed to be tightly bound.^[18]^ It is thus surprising that Cu_3_N nanocrystals have generally poor colloidal stability after purification.

In this work, we aimed at elucidating the precursor conversion mechanism and surface chemistry of Cu_3_N (and Cu_3_PdN) nanocrystals, synthesized from copper nitrate (and palladium acetylacetonate). We first screened the different synthetic methods in the literature, and found the report of Vaughn et al. as the most reproducible.[^12b^, ^19^] We then explored the influence of different parameters on the reaction outcome, including the presence of water, temperature, time, etc. Having obtained the optimal conditions to form phase pure Cu_3_N nanocubes, we then redesigned the purification procedure to yield colloidally stable Cu_3_N nanocrystals. We uncovered the precursor conversion mechanism of the reaction. We found that nitrate and Cu^II^ both oxidize alkylamine to a primary aldimine, forming also Cu^I^. Condensation of the primary aldimine with a second equivalent alkylamine, yields a secondary aldimine and ammonia. The latter is the active nitrogen precursor and reacts with Cu^I^ to Cu_3_N. Finally, the surface of these Cu_3_N and Cu_3_PdN particles has been analyzed. X‐ray photoelectron spectroscopy (XPS) and Fourier Transform Infrared spectroscopy (FTIR) established the presence of carboxylate ligands on the surface, together with amine ligands. Advanced nuclear magnetic resonance (NMR) spectroscopy revealed the dynamics of ligands binding. These fundamental chemistry insights will enable the elucidation of formation mechanisms of other copper‐based colloidal nanocrystals and other metal nitrides.

## Results and Discussion

We decided to optimize the procedure reported by Vaughn et al. by exploring the influence of different parameters.^[12b]^ First, we replaced 1‐octadecene (the solvent) with hexadecane since we reported earlier that 1‐octadecene polymerizes at 240 °C, contaminating the final nanocrystal product and complicating the purification.^[20]^ Concerning reaction temperature, crystalline Cu_3_N was formed between 220 °C and 260 °C (after 15 minutes), with the highest crystallinity for 260 °C (Figure S1). Interestingly, the crystallite size was found to be quite independent of the reaction temperature; 10–11 nm according to the Scherrer analysis of the powder XRD (X‐ray diffraction) reflections. At 200 °C, no particles could be isolated. Furthermore, we took reaction aliquots at 5, 10, 15, 30, and 60 min at 240 °C and 260 °C. Each aliquot was purified and analyzed by transmission electron microscopy (TEM) to observe the nanocrystal growth throughout the reaction. At 240 °C, we observed both small dots and fully formed nanocubes up to 15 min of reaction time (Figures S2). The small particles presumably ripen in the bigger nanocubes since the former disappear at 30 min. After 60 minutes at 240 °C, the XRD showed pure Cu_3_N. By comparison, at 260 °C, the nanocubes were already fully formed after 15 min. After 30 min at 260 °C, the particles started to decompose and crystalline Cu^0^ was formed. Some Cu_2_O could also be detected as a minority phase after full decomposition. (Figure S3).

**Scheme 2:**
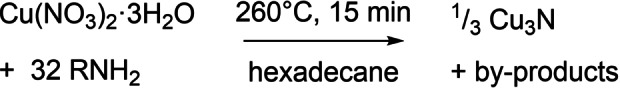
The conditions that lead most reproducibly to phase‐pure Cu_3_N nanocrystals.

The reported procedure has two steps where vacuum is applied. This leads us to investigate the role of water and whether an inert atmosphere is strictly necessary. Note that the precursor contains water (an equivalent of 13 μL for a standard synthesis). We obtained identical Cu_3_N nanocrystals when the vacuum steps were omitted, or, when after applying vacuum (presumably removing water), 13 μL of water was injected into the reaction mixture (Figures S4, S5). However, the same reaction in an open flask did not yield Cu_3_N (or any isolatable material), indicating that an important gaseous intermediate can escape from the reaction mixture. To work under controlled and reproducible conditions, we still choose to perform a short (30 min) degassing step at 50 °C, but it appears that the synthesis is robust against air or water contamination. We also found that both 30 and 60 equivalents of amine ligands yielded Cu_3_N nanocrystals (Figure S6). Based on the above optimization, we arrived at the conditions in Scheme 2.

**Figure 1:**
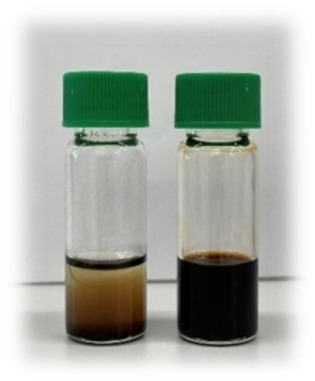
Photograph of Cu_3_N nanocrystals after regular (left) and optimized (right) purification.

After applying the reported precipitation‐and‐redispersion cycles (with ethanol and toluene),^[12b]^ the particles were redispersed in toluene (5 mL), but they precipitated within a couple of minutes, see Figure 1. Since the nanocrystals were colloidally stable in the crude reaction mixture, we hypothesized that the ligands desorbed from the surface during the precipitation‐and‐redispersion cycles. The nature and volume of the solvent used for redispersion play an important role since they determine the position of the adsorption–desorption equilibrium. The ligand–solvent interaction should be favorable enough to disperse the nanocrystals, but not so high that ligands prefer to be fully solvated over being bound to the surface.^[21]^ During purification, it is also preferred to work with quite concentrated dispersions to minimize the loss of nanocrystals over several cycles. On the other hand, too high concentrations might cause impurities to be trapped between the flocculating nanocrystals. Considering the above points, we adapted the purification procedure. Various solvents (toluene, hexane, chloroform, and cyclohexane), as well as non‐solvents (acetone, ethanol, and methanol), were tested. Our final procedure involves two precipitation cycles with acetone (15 mL) followed by a final precipitation with ethanol (15 mL), always redispersing in cyclohexane (5 mL).^[22]^ A 10 v% solution of distilled oleylamine was added after the first acetone wash (1 mL) and after the second acetone wash (2 mL) followed by 5 minutes of sonication. There was no need for a final oleylamine addition after the ethanol wash and the nanocrystals remained colloidally stable. This procedure thus minimized the amount of excess ligand in the final product but provided a stable dispersion (11 mg mL^−1^) of oleylamine‐capped Cu_3_N nanocrystals (Figure 1). From thermogravimetric analysis (TGA), we determined that the dried samples contained 29.2 w % organics (ligands) and the final Cu_3_N yield is 96 % (Figure S7).

Copper palladium nitride (Cu_3_PdN) nanocrystals were also synthesized. The procedure was identical to that of Cu_3_N, except for the reaction temperature (240 °C) and the addition of 0.33 equivalents of palladium(II) acetylacetonate, alongside the Cu(NO_3_)_2_. The doped nanocrystals were purified with the optimized purification method. The synthesis yielded black nanoparticles that were colloidally stable (13 mg mL^−1^). According to TGA, ligands make up 13.22 w% of the dried mass (assuming perfect Cu_3_PdN stoichiometry) and the Cu_3_PdN yield is 91 % (Figure S7).

**Figure 2:**
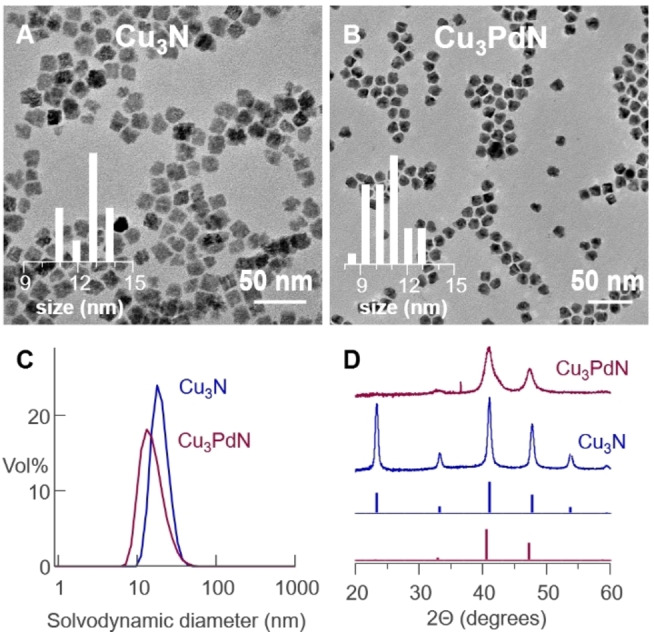
TEM images of A) Cu_3_N and B) Cu_3_PdN. The histograms are shown as an inset. The cube edge length is used as a measure for size. C) DLS and D) powder XRD measurements (the reference XRD reflections are shown as well).

The final dispersions of Cu_3_N and Cu_3_PdN nanocrystals were analyzed with TEM, XRD and dynamic light scattering (DLS) (Figure 2). According to TEM, the average cube edge length is 13.5 nm (*σ*=1.9 nm) for the Cu_3_N nanocubes and 10.2 nm (*σ*=1.4 nm) for the Cu_3_PdN nanocrystals. The solvodynamic diameter obtained from DLS analysis were 17 nm for Cu_3_N and 13 nm for Cu_3_PdN. The sizes obtained via DLS are in line with the sizes obtained via TEM since DLS determines the solvodynamic size of the whole particle, including the surface ligands. Both TEM and DLS support that the dispersions are highly stable since no aggregates are observed. XRD shows quite sharp reflections for Cu_3_N (crystallite size=9.0±1.7 nm), while they are broader for Cu_3_PdN (crystallite size=4.5±1.1 nm). Compared with the sizes obtained from TEM, it is clear that the Cu_3_PdN nanocrystals are polycrystalline. A real space refinement of the Pair Distribution Function further confirms the structure of Cu_3_N and Cu_3_PdN (Figure S8).

**Scheme 3:**
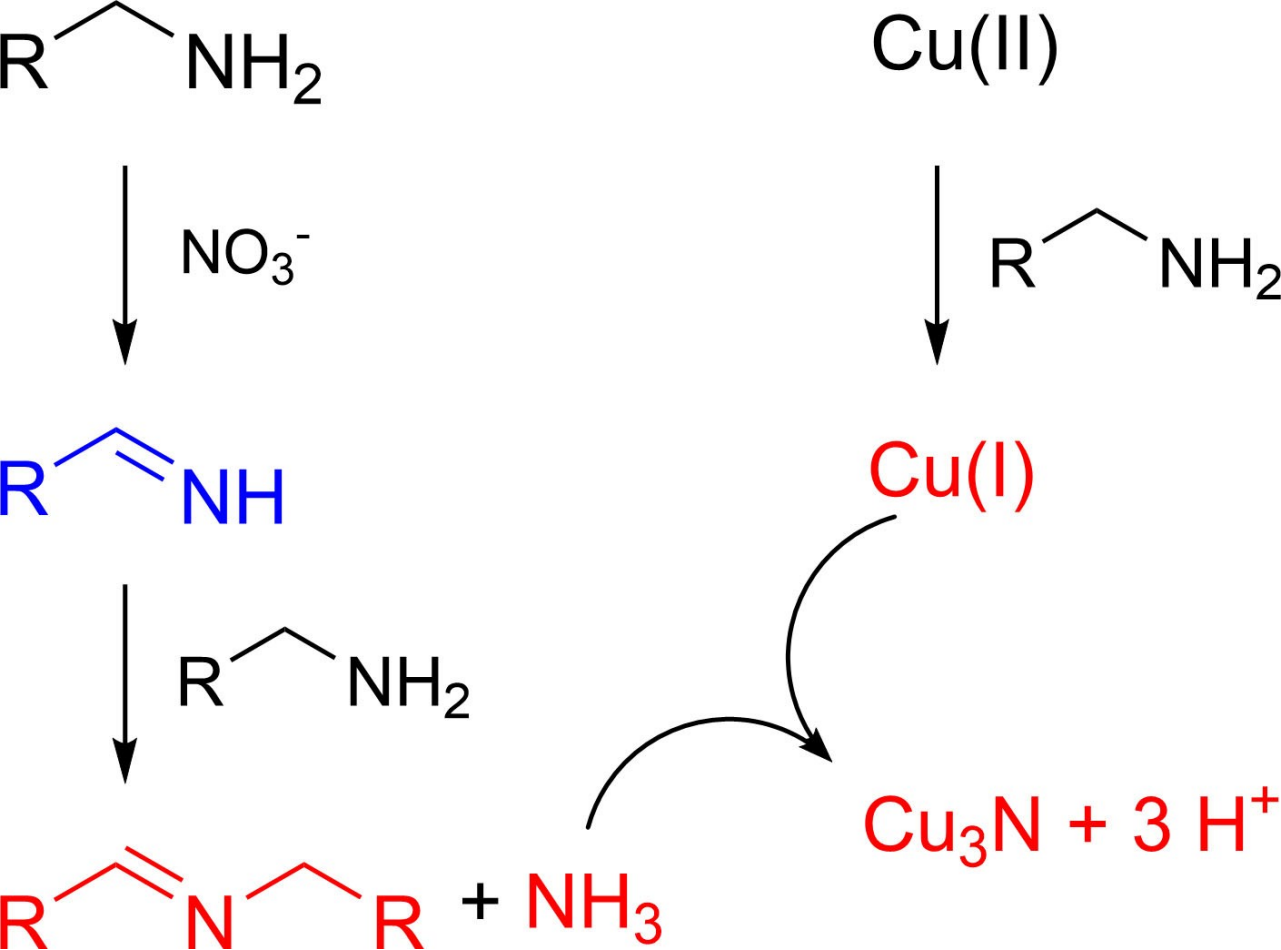
Our proposed pathway for Cu_3_N formation. Precursors are shown in black, detected species in red, and hypothesized intermediates in blue.

Regarding the precursor conversion mechanism, we found the proposed pathways in literature implausible and therefore proposed an alternative hypothesis in which the active nitride source is ammonia, see Scheme 3. First, oleylamine is oxidized to a primary aldimine by nitrate. Nucleophilic addition of a second equivalent of oleylamine forms the more stable, secondary aldimine with the elimination of ammonia. Ammonia reacts with Cu^I^ to Cu_3_N, releasing three protons. The Cu^I^ species was generated by reduction of Cu^II^ by oleylamine upon heating. Cyclic voltammetry confirms a lower reduction potential of Cu^II^ to Cu^I^ upon addition of oleylamine (at room temperature).^[23]^ We hypothesize that the co‐product of this Cu^II^ reduction is the same primary aldimine as mentioned before.

**Figure 3:**
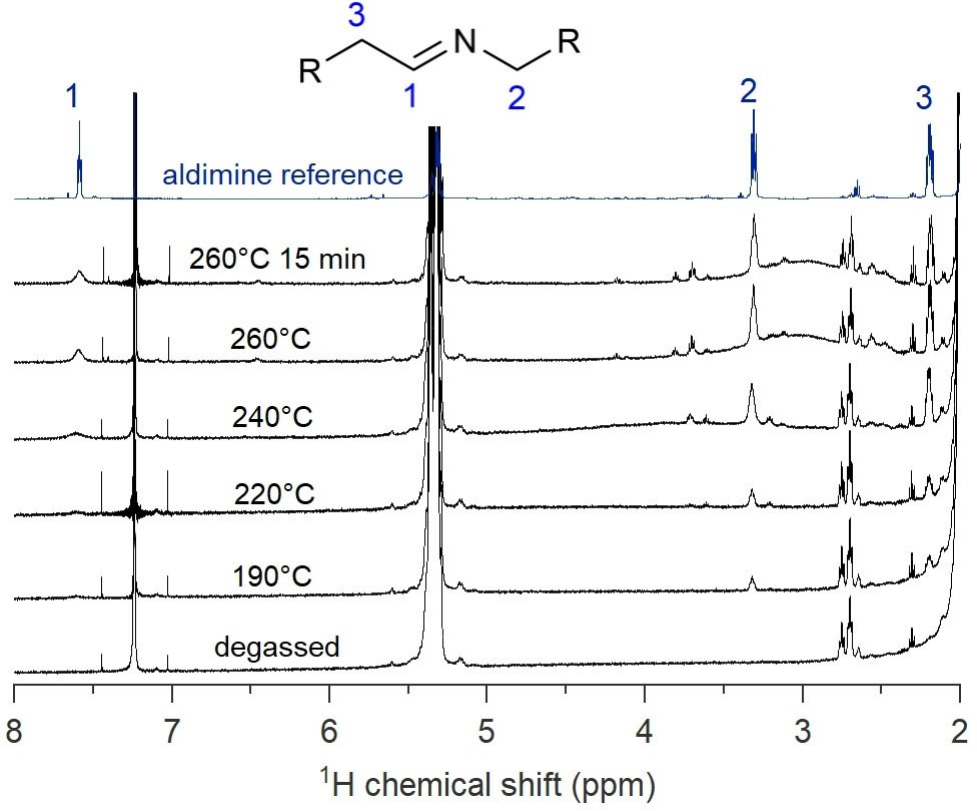
^1^H NMR spectra of aliquots after degassing, at 190 °C (after the color change), at 220 °C, at 240 °C, at 260 °C and at the end of the reaction. Aldimine formation is observed starting from 190 °C. The aldimine reference is shown for comparison.

The copper precursor, Cu(NO_3_)_2_⋅3 H_2_O, does not dissolve in the solvent (hexadecane) until oleylamine is added, indicating the formation of a coordination complex. This is supported by the deep blue color of the reaction mixture, typical for Cu^II^ coordinated by amines.^[24]^ During the heat‐up to 260 °C, the color of the solution changes from blue to yellow around 185 °C, indicating the reduction of Cu^II^ to Cu^I^. Concomitant with the color change, new resonances appear in the ^1^H NMR spectrum of the reaction mixture (Figure 3). We assign these resonances to the secondary aldimine. Upon reaching the reaction temperature (240–260 °C), the reaction mixture turns brown (indicating the formation of Cu_3_N), and we observe in the ^1^H NMR spectrum a significant increase in the aldimine concentration (Figure 3). We confirmed the identity of the aldimine by synthesizing the secondary aldimine of dodecyl aldehyde and octadeylamine, and we found perfect agreement of the resonances **1**–**3**.

Ammonia is detected in the reaction mixture by bubbling Ar through the reaction mixture during synthesis, and dissolving the gasses in either 40 mL or 80 mL water. A commercial ammonia test kit indicates a concentration of 200 mg L^−1^ or 100 mg L^−1^ respectively (Figure S9). Given that the reaction is executed at a scale of 0.24 mmol Cu, and assuming that every nitrate (0.48 mmol) oxidizes one oleylamine molecule, this is the expected amount of ammonia (0.48 mmol, 8 mg). We can also quantify the aldimine formation by using the alkene resonance of oleylamine as the internal standard. For every aldimine, we find 18 oleyl chains, indicating a conversion of 5.5 %. Given that 7.6 mmol oleylamine was used, this amounts to 0.42 mmol aldimine. This value is in reasonable agreement with the amount of ammonia detected (taking into account the error on the ammonia measurement and the fact that oleylamine is only 70 % pure). This quantitative picture thus confirms our hypothesis and indicates a one‐to‐one stoichiometry between nitrate, aldimine, and ammonia. We did not obtain Cu_3_N in this experiment since we removed ammonia from the reaction mixture. This indicates that ammonia is essential in nitride formation. Literature shows that ammonia indeed reacts with copper salts to Cu_3_N.[^11^, ^25^]

**Scheme 4:**
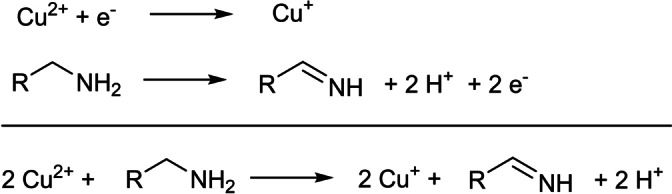
Redox half reactions and overall reaction for the reduction of Cu^2+^ by primary amines.

We also quantified the small amount of aldimine (0.1 mmol) generated during the reduction of Cu^II^ to Cu^I^. This value is about half of the copper amount (0.24 mmol) and can be easily rationalized based on electron counting in the redox reactions. Indeed, the reduction of Cu^II^ to Cu^I^ is a one‐electron process while the oxidation of primary amine to aldimine is a two‐electron process (Scheme 4). Aldimine has also been observed in the synthesis of Cu^0^ and Pd^0^ nanocrystals,^[26]^ and it appears to be the typical oxidation product of oleylamine. Scheme 4 also makes clear that the oxidation of primary amine generates a large amount of protons which are presumably absorbed by the excess oleylamine. These protons are observed in the ^1^H NMR spectrum as a broad resonance around 3 ppm (see Figure 3). They do not appear in the typical region for alkylammonium resonances, since maximally 1.2 mmol protons are generated and oleylamine is still present in excess (7 mmol). Given that proton equilibria are typically fast, we thus observe the population averaged chemical shift between protonated oleylamine and unprotonated oleylamine.

**Figure 4:**
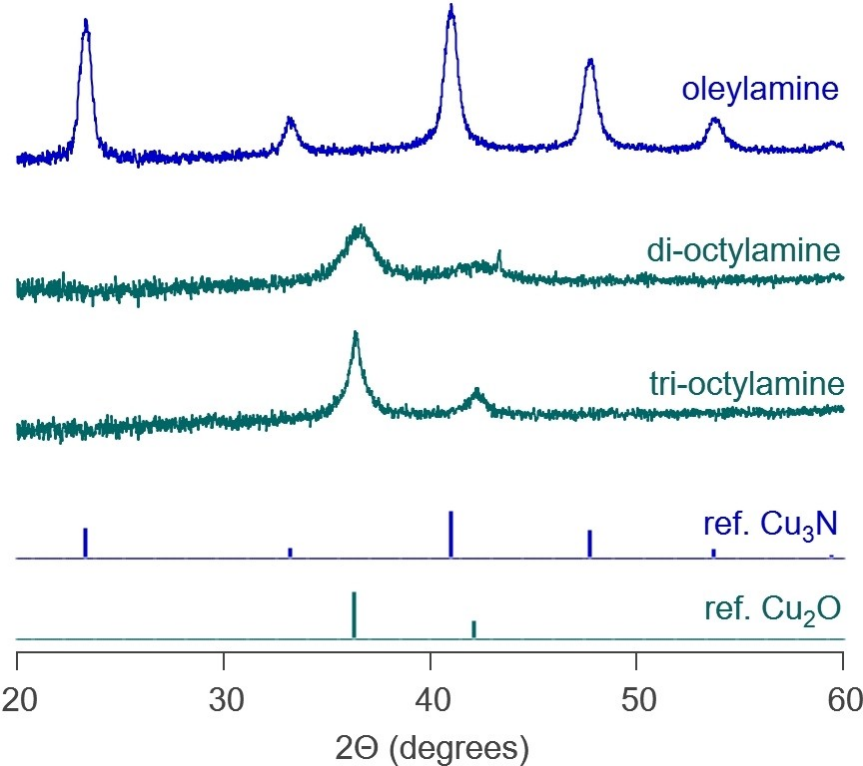
XRD spectra of the syntheses with different amines. The reference of bulk Cu_3_N (blue) and bulk Cu_2_O (green) are shown.

Our proposed pathway requires a primary amine. To further test our hypothesis, we thus attempted the synthesis of Cu_3_N with dioctylamine and trioctylamine. As expected, the reaction did not produce Cu_3_N but Cu_2_O instead (Figure 4). Also in the NMR spectrum, we do not find aldimine in the case of trioctylamine and only a very small amount in the case of dioctylamine. The latter can be directly oxidized to a secondary aldimine, without a primary aldimine intermediate (and thus without ammonia elimination) (Figure S10). As an additional control, we performed a reaction with copper nitrate and dioctylamine, and bubbled ammonia through the reaction mixture as soon as Cu^I^ is formed. XRD analysis shows that Cu_3_N is formed during this reaction along with some Cu_2_O (Figure S11). This control experiment suggests that our proposed pathway—Cu^I^ reacting with in situ produced ammonia—is plausible.

We thus firmly established that nitrate is not the nitrogen source for Cu_3_N but rather oxidizes the amine to aldimine. Cu_3_PdN nanocrystals follows the same mechanism based on the formation of aldimine and ammonia during the reaction (Figure S12). The mechanism is reminiscent of the one for InN, where In^3+^ oxidizes primary amines into aldimine and nucleophilic attack of lithium oleylamide generates amide, NH_2_^−^.^[27]^ However, an important difference is that for copper, an additional oxidant (nitrate) is required. The reduction of Cu^2+^ alone does not seem to generate sufficient ammonia to form copper nitride since other copper salts do not generate copper nitride.^[12c]^ Or perhaps it generates ammonia at too low temperatures, where the formation of Cu_3_N is not yet favored. More detailed insight into the crystallization mechanism of Cu_3_N could shed light on this issue.

**Figure 5:**
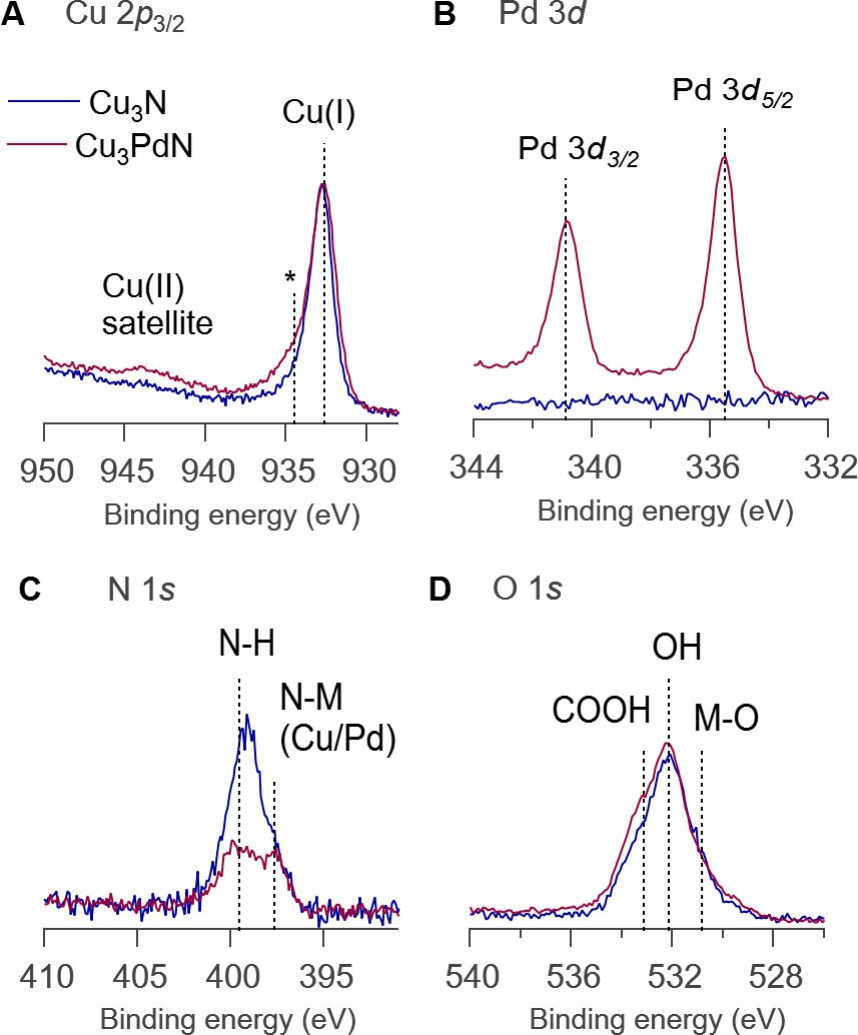
XPS core level spectra of Cu_3_N and Cu_3_PdN, including A) Cu 2*p*_3/2_, B) Pd 3*d*, C) N 1*s*, and D) O 1*s*.

X‐ray photoelectron spectroscopy (XPS) was used to explore the surface chemistry. Survey spectra for both compounds (Figure S13) show spectral signatures from all expected elements. In addition, a small amount of Si was detected most likely from the silicone grease used during the synthesis. The binding energy (BE) position of the main feature in the Cu 2*p*_3/2_ core level (932.7±0.1 eV) is commensurate with the expected Cu^I^ oxidation state for both samples (Figure 5A). In addition, both samples show a shoulder towards the higher BE of the main peak (marked with an asterisk in Figure 5A) and in the Cu_3_PdN sample, a clear Cu^II^ satellite is also visible. From peak fit analysis (Figure S14), the contribution of these additional chemical states relative to the main Cu^I^ line is 12.3±0.5 rel. at.% for Cu_3_N and 17.9±0.5 rel.at.% for Cu_3_PdN, respectively. The Pd 3*d* core level (Figure 5B) is only observed for the Cu_3_PdN sample. The BE of the Pd 3*d*_5/2_ component (335.5±0.1 eV) is commensurate with Pd^0^ or Pd^I^ with a spin‐orbit‐splitting of the doublet of 14.6 eV. In both samples, the N 1*s* core level (Figure 5C) displays two contributions. The lower BE feature at 397.8±0.2 eV corresponds to metal‐nitride and the higher BE feature at 399.6±0.2 eV corresponds to the expected oleylamine ligand, bound to the nanocrystal surface. The O 1*s* core level (Figure 5D) shows three discernible contributions from metal oxide, metal hydroxide, and carboxylate environments. Together with the Cu^II^ species, we infer that the surface of the particles contains an amorphous copper oxyhydroxide layer. The carboxylate is assigned to a surface bound oleate ligand and its presence is also detected by FTIR (broad signal at 1577 cm^−1^, see Figure S15). Oleate is most likely formed by oxidation of oleylamine by nitrate, as we reported earlier in the synthesis of cerium oxide nanocrystals.^[15b]^

**Figure 6:**
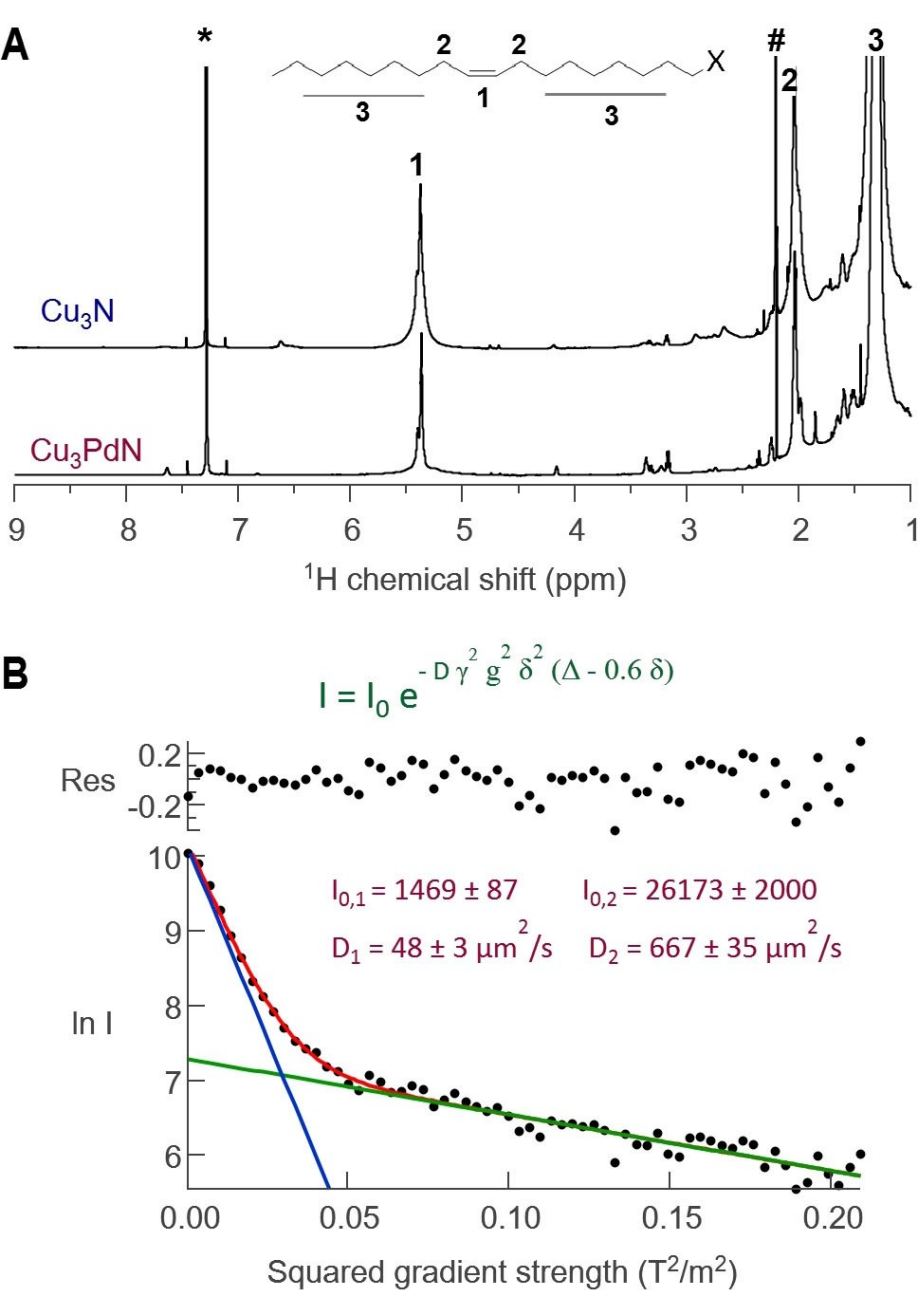
Nuclear magnetic resonance spectroscopy of the purified Cu_3_N and Cu_3_PdN NCs. A) ^1^H NMR spectrum in chloroform‐*d* of the NCs indicating the presence of an oleyl chain. B) Diffusion decay of the alkene region of the Cu_3_PdN NCs showing a slow and a fast diffusing species. The residuals of the fit are also shown.

The ^1^H NMR spectra of Cu_3_N and Cu_3_PdN show the typical signature of an oleyl chain (Figure 6A). While the alkene resonance is reasonably sharp, the resonances close to the binding group are indistinguishable from the background. This broadening is likely due to fast T_2_ relaxation induced by copper.^[18b]^ Based on the line width of the alkene resonance,^[28]^ we inferred that the ligand is dynamic and in fast exchange between a bound and free state. This is confirmed by a relatively high diffusion coefficient (*D*=174 μm^2^ s^−1^) determined by pulsed field gradient experiments on the Cu_3_N dispersion (Figure S16). For Cu_3_PdN, we noticed a second, very broad alkene resonance of low intensity underneath the sharp signal. A careful pulsed field gradient experiment revealed that the alkene resonance is a superposition of two species, one diffusing with *D*=48 μm^2^ s^−1^ and the other one with *D*=667 μm^2^ s^−1^ (Figure 6 B). The small diffusion coefficient corresponds to a solvodynamic diameter of 17 nm (via the Stokes–Einstein equation), which agrees quite well with a nanocrystal of 12 nm and a ligand shell of 2 nm thickness. We thus assign the broad resonance to tightly bound ligand, presumably the oleate. The larger diffusion constant corresponds most likely to oleylamine. We could only detect the tightly bound oleate in the Cu_3_PdN sample, but oleate is also present on the surface of Cu_3_N according to our XPS results, albeit to a lesser extent. Unfortunately, all NMR signals appear more broadened in the Cu_3_N sample and the tightly bound oleate could not be detected in NMR.

**Figure 7:**
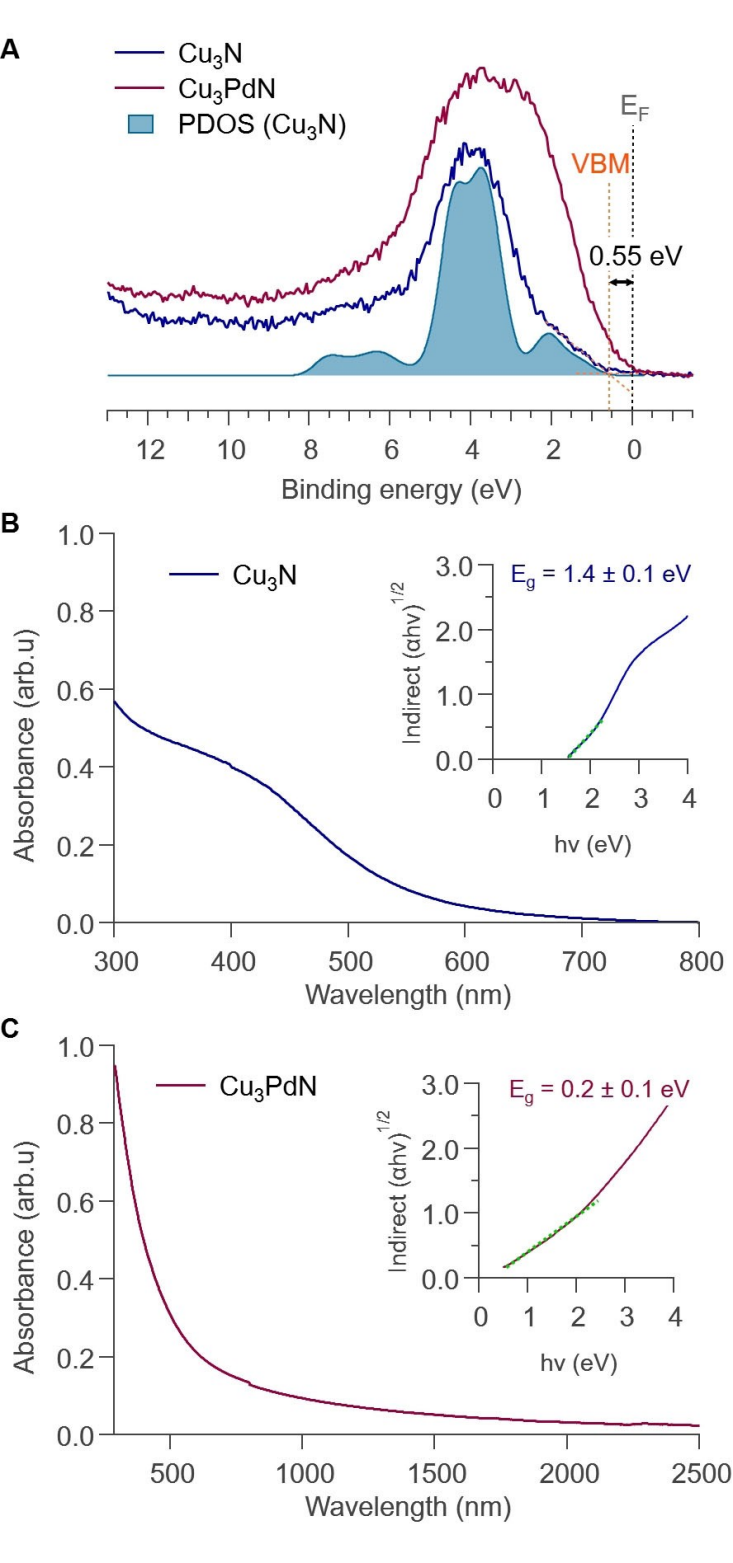
A) Valence region of Cu_3_N and Cu_3_PdN, including XPS valence spectra and the broadened and cross‐section‐weighted sum of the projected density of states (PDOS) from density functional theory (DFT). The position of the valence band maximum (VBM) of Cu_3_N and the position of the Fermi energy (*E*_F_) are also shown. B), C) UV/Vis absorption spectra of Cu_3_N in cyclohexane and Cu_3_PdN in tetrachloroethylene with their corresponding Tauc plots as an inset, respectively. Indirect band gaps were determined from linear fitting to the low energy region of the Tauc plots (shown in dotted green line).

In addition to the identification of the chemical species present in the samples, XPS was also used to probe the electronic structure of Cu_3_N and Cu_3_PdN by collecting valence band spectra. Figure 7A shows the valence band spectra of the two compounds, as well as the broadened and cross section weighted total density of states of Cu_3_N from density functional theory (DFT) calculations. The experimental and theoretical results for Cu_3_N agree very well. The projected density of states calculations (Figure S17) show that the valence band of Cu_3_N is dominated by Cu 3*d* states with only minor mixing of Cu 3*p* states and minimal nitrogen contributions. The valence band maximum (VBM) position of Cu_3_N is 0.55±0.05 eV from the Fermi energy *E*_F_. In comparison, the valence spectrum of Cu_3_PdN shows additional intensity towards the *E*_F_ from Pd states closing the VBM‐*E*_F_ gap. In parallel, UV/Vis has been used to determine the optical band gap of the particles. An indirect band gap has been reported for Cu_3_N thin films according to theoretical band structure calculations.^[2]^ To our knowledge, the type of optical band gap (direct or indirect) has not yet been reported for Cu_3_PdN. In this work, we assumed an indirect optical band gap for both samples. A Tauc plot analysis resulted in an optical band gap of 1.4±0.1 eV and 0.2±0.1 eV for our Cu_3_N and Cu_3_PdN NCs, respectively (Figure 7B and C). The reduction in optical band gap is commensurate with the closing of the electronic VBM‐*E*_F_ separation observed in XPS.

## Conclusion

In conclusion, we successfully optimized the synthesis of Cu_3_N and Cu_3_PdN nanocrystals in order to obtain phase pure, colloidally stable nanocrystals via modulating the reaction parameters with a focus on the purification. We provided experimental support for a precursor conversion pathway that hypothesized ammonia as the active nitrogen source. We proposed that primary aldimine is the oxidation product of the ligand (oleylamine), oxidized by both Cu^II^, and nitrate. Nucleophilic addition and elimination of a second oleylamine molecule onto the primary aldimine released ammonia, which subsequently reacted with Cu^I^ to form Cu_3_N and Cu_3_PdN. The surface of the nanocrystals was capped by a mixture of oleylamine and oleate. The latter was formed in situ from the further oxidation of aldimine. The addition of palladium to copper nitride reduces the optical band gap as well as the separation between the valence band maximum (VBM) and Fermi energy *E*_F_.

## Conflict of interest

The authors declare no conflict of interest.

1

## Supporting information

As a service to our authors and readers, this journal provides supporting information supplied by the authors. Such materials are peer reviewed and may be re‐organized for online delivery, but are not copy‐edited or typeset. Technical support issues arising from supporting information (other than missing files) should be addressed to the authors.

Supporting Information

## Data Availability

The data that support the findings of this study are openly available in Zenodo at https://doi.org/10.5281/zenodo.6542908.

## References

[1a] Z. Chen , C. Sun , W. Guo , Z. Chen in Nonmagnetic and Magnetic Quantum Dots (Ed.: V. N. Stavrou ), IntechOpen, Rijeka, 2017;

[1b] Y. Ma , L. Xiong , Y. Lu , W. Zhu , H. Zhao , Y. Yang , L. Mao , L. Yang , Front. Chem. 2021, 9, 638216; 34307294 10.3389/fchem.2021.638216 PMC8299337

[1c] M. Parvizian , J. De Roo , Nanoscale 2021, 13, 18865–18882. 34779811 10.1039/d1nr05092c PMC8615547

[2] A. Zakutayev , C. M. Caskey , A. N. Fioretti , D. S. Ginley , J. Vidal , V. Stevanovic , E. Tea , S. Lany , J. Phys. Chem. Lett. 2014, 5, 1117–1125. 26274458 10.1021/jz5001787

[3] T. Maruyama , T. Morishita , Appl. Phys. Lett. 1996, 69, 890–891.

[4a] Z. Yin , C. Yu , Z. Zhao , X. Guo , M. Shen , N. Li , M. Muzzio , J. Li , H. Liu , H. Lin , J. Yin , G. Lu , D. Su , S. Sun , Nano Lett. 2019, 19, 8658–8663; 31682758 10.1021/acs.nanolett.9b03324

[4b] C. Panda , P. W. Menezes , M. Zheng , S. Orthmann , M. Driess , ACS Energy Lett. 2019, 4, 747–754;

[4c] P. X. Xi , Z. H. Xu , D. Q. Gao , F. J. Chen , D. S. Xue , C. L. Tao , Z. N. Chen , RSC Adv. 2014, 4, 14206–14209.

[5a] D. Bocharov , A. Anspoks , J. Timoshenko , A. Kalinko , M. Krack , A. Kuzmin , Radiat. Phys. Chem. 2020, 175, 108100–108104;

[5b] G. Paniconi , Z. Stoeva , H. Doberstein , R. I. Smith , B. L. Gallagher , D. H. Gregory , Solid State Sci. 2007, 9, 907–913.

[6a] X. Y. Cui , A. Soon , A. E. Phillips , R. K. Zheng , Z. W. Liu , B. Delley , S. P. Ringer , C. Stampfl , J. Magn. Magn. Mater. 2012, 324, 3138–3143;

[6b] H. Jacobs , U. Zachwieja , J. Less-Common Met. 1991, 170, 185–190.

[7a] U. Hahn , W. Weber , Phys. Rev. B 1996, 53, 12684–12693; 10.1103/physrevb.53.12684 9982940

[7b] F. Gulo , A. Simon , J. Kohler , R. K. Kremer , Angew. Chem. Int. Ed. 2004, 43, 2032–2034; 10.1002/anie.200353424 15065294

[8] J. Choi , E. G. Gillan , Inorg. Chem. 2005, 44, 7385–7393. 16212364 10.1021/ic050497j

[9a] U. Zachwieja , H. Jacobs , J. Less-Common Met. 1990, 161, 175–184;

[9b] R. Juza , H. Hahn , Z. Anorg. Allg. Chem. 1939, 241, 172–178.

[10] R. Deshmukh , G. B. Zeng , E. Tervoort , M. Staniuk , D. Wood , M. Niederberger , Chem. Mater. 2015, 27, 8282–8288.

[11] A. Egeberg , L. Warmuth , S. Riegsinger , D. Gerthsen , C. Feldmann , Chem. Commun. 2018, 54, 9957–9960. 10.1039/c8cc04893b 30116819

[12a] H. Wu , W. Chen , J. Am. Chem. Soc. 2011, 133, 15236–15239; 21894995 10.1021/ja204748u

[12b] D. D. Vaughn Ii , J. Araujo , P. Meduri , J. F. Callejas , M. A. Hickner , R. E. Schaak , Chem. Mater. 2014, 26, 6226–6232;

[12c] D. Wang , Y. Li , Chem. Commun. 2011, 47, 3604–3606; 10.1039/c0cc04902f 21321695

[12d] R. K. Sithole , L. F. E. Machogo , M. J. Moloto , S. S. Gqoba , K. P. Mubiayi , J. Van Wyk , N. Moloto , J. Photochem. Photobiol. A 2020, 397, 112577–112587.

[13] R. Kadzutu-Sithole , L. F. E. Machogo-Phao , T. Kolokoto , M. Zimuwandeyi , S. S. Gqoba , K. P. Mubiayi , M. J. Moloto , J. Van Wyk , N. Moloto , RSC Adv. 2020, 10, 34231–34246. 35519021 10.1039/c9ra09546b PMC9056776

[14] C. E. Housecroft , A. G. Sharpe , Inorganic Chemistry, 5th *ed*., Pearson, Upper Saddle River, 2018.

[15a] K. De Keukeleere , S. Coucke , E. De Canck , P. Van Der Voort , F. Delpech , Y. Coppel , Z. Hens , I. Van Driessche , J. S. Owen , J. De Roo , Chem. Mater. 2017, 29, 10233–10242;

[15b] M. Calcabrini , D. Van den Eynden , S. S. Ribot , R. Pokratath , J. Llorca , J. De Roo , M. Ibanez , JACS Au 2021, 1, 1898–1903. 35574040 10.1021/jacsau.1c00349 PMC8611721

[16] M. A. Boles , D. Ling , T. Hyeon , D. V. Talapin , Nat. Mater. 2016, 15, 141–153. 26796733 10.1038/nmat4526

[17a] B. Fritzinger , I. Moreels , P. Lommens , R. Koole , Z. Hens , J. C. Martins , J. Am. Chem. Soc. 2009, 131, 3024–3032; 19199431 10.1021/ja809436y

[17b] N. C. Anderson , P. E. Chen , A. K. Buckley , J. De Roo , J. S. Owen , J. Am. Chem. Soc. 2018, 140, 7199–7205; 29746124 10.1021/jacs.8b02927

[17c] C. Grote , K. J. Chiad , D. Vollmer , G. Garnweitner , Chem. Commun. 2012, 48, 1464–1466. 10.1039/c1cc14630k 22057005

[18a] R. Dierick , F. Van den Broeck , K. De Nolf , Q. Zhao , A. Vantomme , J. C. Martins , Z. Hens , Chem. Mater. 2014, 26, 5950–5957;

[18b] A. Oliva-Puigdomènech , J. De Roo , J. Kuhs , C. Detavernier , J. C. Martins , Z. Hens , Chem. Mater. 2019, 31, 2058–2067.

[19] R. W. Lord , C. F. Holder , J. L. Fenton , R. E. Schaak , Chem. Mater. 2019, 31, 4605–4613.

[20] E. Dhaene , J. Billet , E. Bennett , I. Van Driessche , J. De Roo , Nano Lett. 2019, 19, 7411–7417. 31525055 10.1021/acs.nanolett.9b03088

[21] J. Zito , I. Infante , Acc. Chem. Res. 2021, 54, 1555–1564. 33635646 10.1021/acs.accounts.0c00765 PMC8028043

[22] D. Doblas , T. Kister , M. Cano-Bonilla , L. Gonzalez-Garcia , T. Kraus , Nano Lett. 2019, 19, 5246–5252. 31251877 10.1021/acs.nanolett.9b01688

[23] L. Castilla-Amorós , D. Stoian , J. R. Pankhurst , S. B. Varandili , R. Buonsanti , J. Am. Chem. Soc. 2020, 142, 19283–19290. 33135885 10.1021/jacs.0c09458

[24a] F. Cui , Y. Yu , L. Dou , J. Sun , Q. Yang , C. Schildknecht , K. Schierle-Arndt , P. Yang , Nano Lett. 2015, 15, 7610–7615; 26496181 10.1021/acs.nanolett.5b03422

[24b] S. Jeong , Y. Liu , Y. Zhong , X. Zhan , Y. Li , Y. Wang , P. M. Cha , J. Chen , X. Ye , Nano Lett. 2020, 20, 7263–7271. 32866022 10.1021/acs.nanolett.0c02648

[25] T. Nakamura , H. Hayashi , T. A. Hanaoka , T. Ebina , Inorg. Chem. 2014, 53, 710–715. 24369736 10.1021/ic4011604

[26a] R. W. Man , A. R. Brown , M. O. Wolf , Angew. Chem. Int. Ed. 2012, 51, 11350–11353; 10.1002/anie.201205057 23011969

[26b] F. Yarur Villanueva , P. B. Green , C. Qiu , S. R. Ullah , K. Buenviaje , J. Y. Howe , M. B. Majewski , M. W. B. Wilson , ACS Nano 2021, 15, 18085–18099. 10.1021/acsnano.1c06730 34705409

[27] Y. Chen , N. T. Landes , D. J. Little , R. Beaulac , J. Am. Chem. Soc. 2018, 140, 10421–10424. 30081636 10.1021/jacs.8b06063

[28] J. De Roo , N. Yazdani , E. Drijvers , A. Lauria , J. Maes , J. S. Owen , I. Van Driessche , M. Niederberger , V. Wood , J. C. Martins , I. Infante , Z. Hens , Chem. Mater. 2018, 30, 5485–5492.

