# Supplementary material for: The Chemistry of Cu3N and Cu3PdN Nanocrystals
Source: Angew Chem Int Ed Engl. 2022 Jun 15;61(31):e202207013. doi: 10.1002/anie.202207013 (PMC9400990; doi:10.1002/anie.202207013)
Supplement: Supplementary file 1 — Supporting Information [file ANIE-61-0-s001.pdf]

Angewandte Chemie
Eine Zeitschrift der Gesellschaft Deutscher Chemiker

Supporting Information

The Chemistry of Cu_3N and Cu_3PdN Nanocrystals

*M. Parvizian, A. Duràn Balsa, R. Pokratath, C. Kalha, S. Lee, D. Van den Eynden, M. Ibáñez, A. Regoutz, J. De Roo**

Experimental Section

Materials. For the standard copper nitride and copper palladium doped nitride nanoparticles, copper nitrate ($Cu(NO_3)_2 \cdot 3H_2O$, STREM Chemicals, 99.5 %) and distilled oleylamine ($C_{18}H_{35}NH_2$, Sigma-Adrich, 70 %) were used as precursors in hexadecane ($C_{16}H_{34}$, Sigma Aldrich 99 %). Palladium(II) 2,4- pentadionate ($C_{10}H_{14}O_4Pd$, Alfa Aesar, Pd 34.7 %) was used for doping. For the mechanistic investigations, various precursors were used: copper chloride ($CuCl_2 \cdot 2\ H_2O$, Sigma-Adrich, ≥99.0%), dioctylamine ($CH_3(CH_2)_7NH\text{-}(CH_2)_7CH_3$), Sigma-Aldrich, 97 %), trioctylamine ($(CH_3(CH_2)_7)_3N$, Sigma-Aldrich, 98 %). For the aldimine synthesis, lauric aldehyde ($CH_3(CH_2)_{10}CHO$, Sigma-Aldrich, ≥ 95 %) was used. Cyclohexane, absolute ethanol and acetone were used for the purification step. Deuterated chloroform ($CDCl_3$, 98 %) was used for NMR study.

Synthesis of Cu_3N. The synthesis for Cu_3N nanocrystals was adapted from the literature.[1] Copper nitrate (0.24 mmol, 60 mg, 1 eq) was dissolved in 7.5 mL of hexadecane in a three-neck flask and stirred. Distilled oleylamine (7.57 mmol, 2.5 mL, 31 eq) was added and the mixture was degassed for 30 minutes at 50 °C. Under argon, the temperature was increased to 260 °C at a rate of 10 °C per minute. The mixture was left to react for 15 minutes. The flask was then removed from the heating mantle and left to cool to room temperature over the course of an hour. The 10 mL solution of nanoparticles were transferred to centrifuge tubes. A total of 15 mL of acetone was added and the particles were centrifuged at 5 000 rpm for 3 minutes. The supernatant was removed and the particles were redispersed in 4 mL of cyclohexane. A 10% by volume stock solution (SS) of purified oleylamine in cyclohexane was prepared. Oleylamine (1 mL SS) was added to the redispersed particles, so the total redispersion volume was 5 mL. The particles were placed in the ultrasonic bath until fully redispersed. The particles were washed once more with acetone (15 mL) and this time redispersed in 3 mL of cyclohexane. 2 mL of the oleylamine SS was added, so the volume was still 5 mL. The particles were placed in the ultrasonic bath. 15 mL of ethanol was added for a final wash and the particles were centrifuged at 8’000 rpm for 10 minutes. The supernatant was removed, and the particles were redispersed in 2 mL of cyclohexane.

Synthesis of Cu_3PdN. The synthesis for Cu_3PdN nanocrystals was adapted from the literature.[1] Copper nitrate (0.24 mmol, 60 mg, 1 eq) was dissolved in 7.5 mL of hexadecane in a three-neck flask and stirred. Palladium(II) 2,4- pentadionate (0.08 mmol, 25.2 mg, 0.33 eq) was added and stirred. Distilled oleylamine (7.57 mmol, 2.5 mL, 31 eq) was added and the mixture was degassed for 30 minutes at 50 °C. Under argon, the temperature was increased to

240°C at a rate of 10°C per minute. The mixture was left to react for 15 minutes. The flask was removed from the heating mantle and left to cool to room temperature over the course of an hour. The nanocrystals were purified as described previously. However, contrary to the Cu_3N nanoparticles, the final centrifugation after the ethanol wash required 10 - 15 minutes at 10,000 rpm to precipitate the nanoparticles.

Synchrotron X-ray total scattering experiments. Samples were prepared in 1mm polyamide kapton tube. The samples were measured at beamline P21.1 at DESY in Hamburg, Germany. X-ray total scattering data were collected at room temperature in rapid acquisition mode, using a Perkin Elmer digital X-ray flat panel amorphous silicon detector (2048 × 2048 pixels and 200 × 200 μm pixel size) with a sample-to-detector distance of 380 mm. The incident wavelength of the X-rays was λ = 0.1220 Å (101.62 keV). Calibration of the experimental setup was performed using a Ni standard.

Raw 2D data were corrected for geometrical effects and polarization, then azimuthally integrated to produce 1D scattering intensities versus the magnitude of the momentum transfer Q (where $Q = 4\pi \sin \theta/\lambda$ for elastic scattering) using pyFAI and xpdtools.[2] The program xPDFsuite with PDFgetX3 was used to perform the background subtraction, further corrections, and normalization to obtain the reduced total scattering structure function $F(Q)$, and Fourier transformation to obtain the pair distribution function (PDF), $G(r)$.[3] For data reduction, the following parameters were used after proper background subtraction: Q_{min} = 0.8 $Å^{-1}$, Q_{max} = 19 $Å^{-1}$, Rpoly = 0.9 Å. Modeling and fitting were carried out using Diffpy-CMI or PDFgui.[4]

XPS and DFT. X-ray photoelectron spectroscopy (XPS) measurements were performed on a Thermo Scientific K-Alpha spectrometer with a monochromated microfocused Al Kα X-ray source (hυ = 1486.7 eV) and a spot size of 400 μm. The X-ray source was operated at 6 mA emission current and 12 kV anode bias and a flood gun was used for charge compensation. A pass energy of 20 eV was used for all core level and valence spectra. The Thermo Scientific Avantage software package was used for all data analysis. All XPS core level spectra were normalized to the peak height of the main feature in the Cu $2p_{3/2}$ spectra.

The details of the density functional theory (DFT) calculations for Cu_3N, including density of states, have been previously reported.[5] [6] [7] To enable the direct comparison of the theory with the experimental valence spectra, the calculated projected density of states (PDOS) was broadened with a Gaussian to match the experimental broadening of 600 meV. The PDOS was weighted by one-electron photoionisation cross sections for the respective orbitals of Cu and N using the Galore software package, based on Scofield cross section values. [8]

NMR measurements. NMR measurements were recorded at 298K on Bruker UltraShield 500 MHz spectrometer or a 600 MHz Bruker Avance III spectrometer. Regular ^{1}H NMR measurement were acquired with a 30 degree pulse with a recycle delay of 1.5 sec. Quantitative ^{1}H NMR measurements were acquired with a 90 degree pulse, 64k data points, 20 ppm spectral width, and a recycle delay of 30 sec. DOSY measurements were performed with a double stimulated echo and bipolar gradient pulses (dstebpgp2s). The gradient strength was varied quadratically from 2-95% of the probe’s maximum value in 16 or 64 steps. The gradient pulse duration and diffusion delay were optimized to ensure a final attenuation of the signal in the final increment of less than 10% relative to the first increment. The diffusion coefficients were obtained by fitting a modified Stejskal-Tanner equation to the signal intensity decay:

$$I = I_0 e^{-(\gamma\delta g)^2 D(\Delta - 0.6\delta)}$$

I are the signal intensities, D are the linear diffusion coefficients, γ is the gyromagnetic ratio of the studied nucleus, g is the gradient strength, δ is the pulsed field gradient duration and Δ is the diffusion delay. A correction factor of 0.6 is applied for δ due to the smoothed squared pulse shape used for the gradient pulses.[9]

Other instrumentation. TEM imaging was done using a JEOL JEM2800 field emission gun microscope operated at 200 kV equipped with a TVIPS XF416ES TEM camera. DLS measurements were conducted on a Malvern Zetasizer Ultra in backscattering mode (173°) in a glass cuvette. All measurements were performed at 25°C after equilibrating inside the system for 240 seconds, sample concentration was tuned to achieve system attenuator values between 9-10. UV-VIS spectra were recorded on a PerkinElmer Lambda 365. FTIR spectra were recorded on a Perkin Elmer Spectrum Two spectrometer (attenuated total reflection, ATR).

Supporting figures

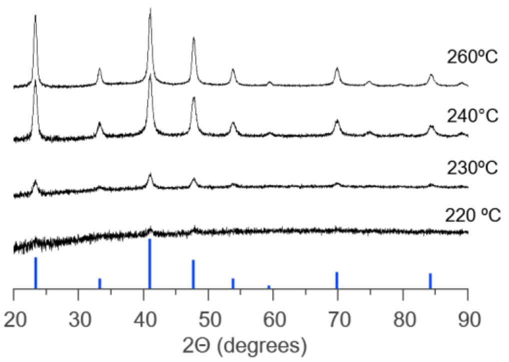

Figure S1. Powder XRD of Cu_3N was obtained after 15 min at the different reaction temperatures. Bulk Cu_3N reference is presented in blue.

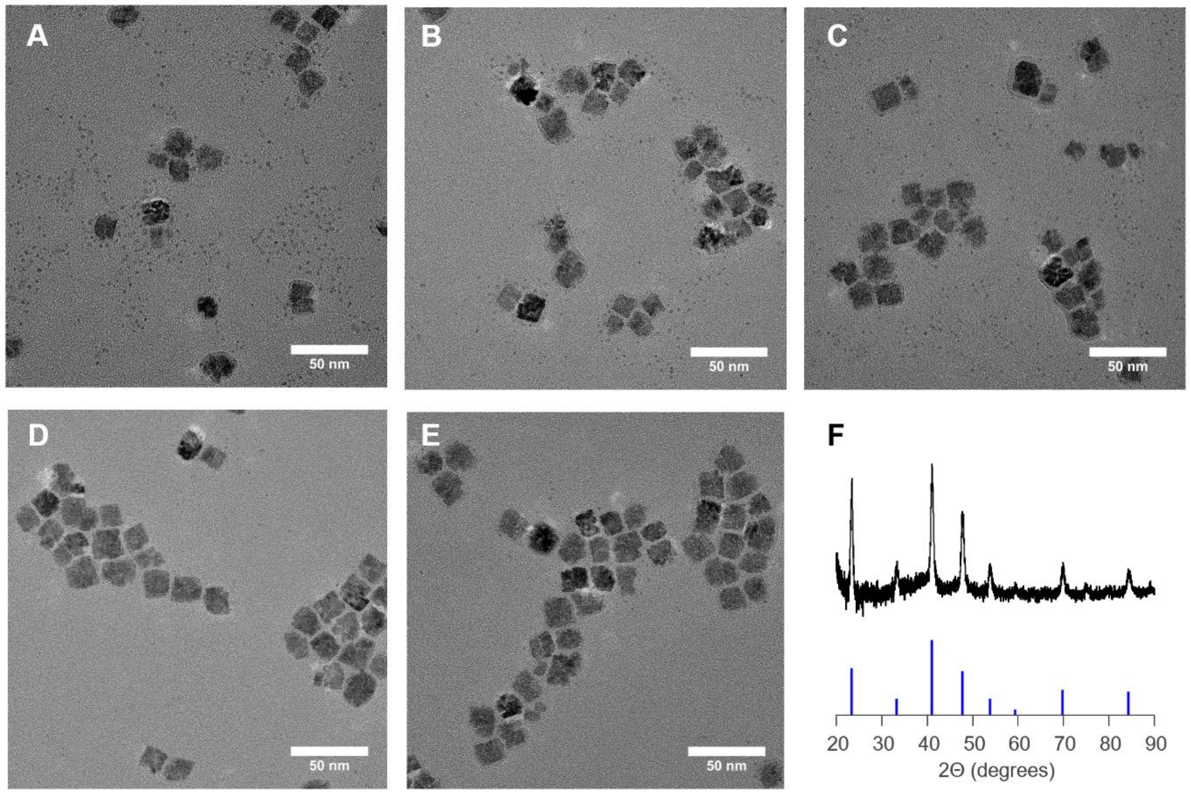

Figure S2. TEM images from aliquots at 5(A), 10 (B), 15 (C), 30 (D) and 60 (E) min at 240°C. The XRD of the final phase at 60 min is shown corresponding to pure Cu_3N. No decomposition is observed even after 60 min. XRD of bulk Cu_3N is shown as a reference (D).

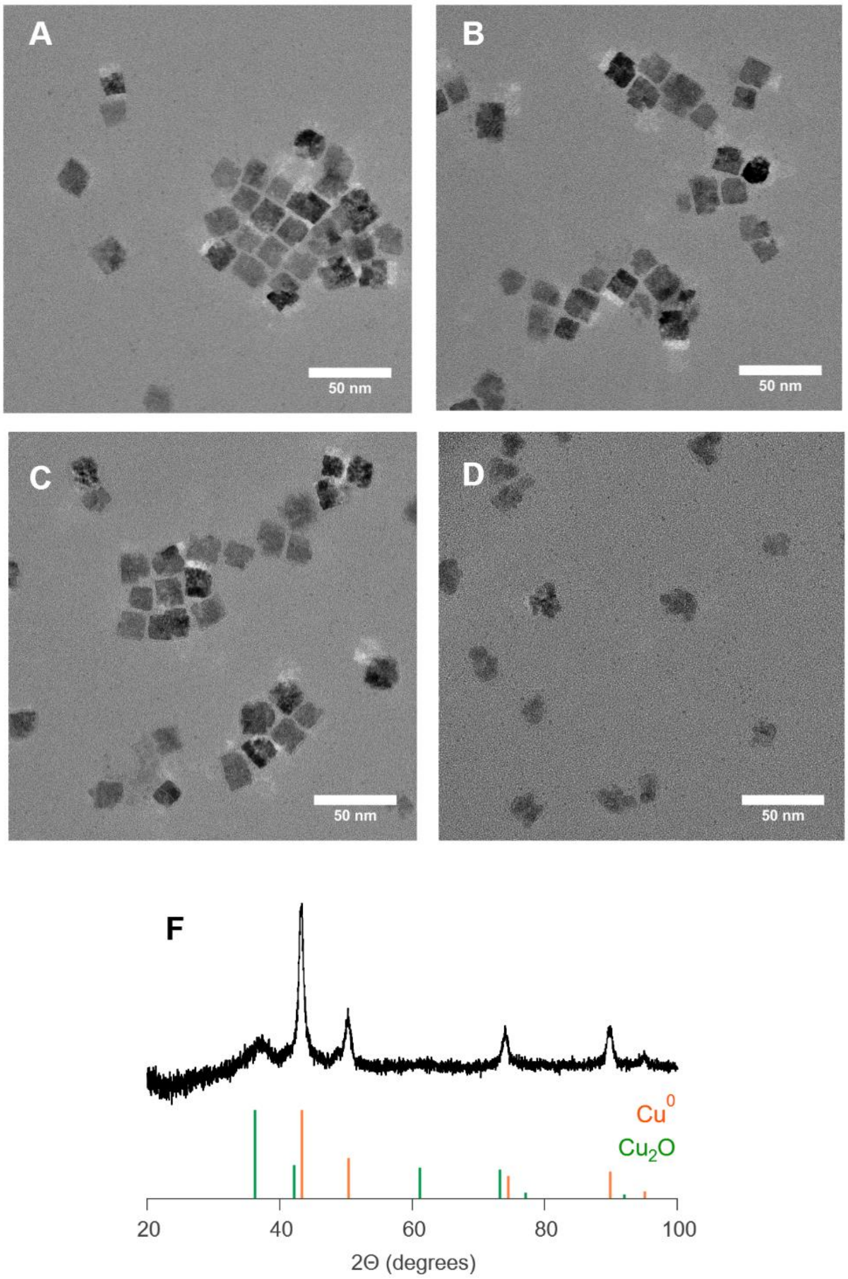

Figure S3. TEM images from aliquots at 5(A), 10 (B), 15 (C), and 30 (D) min at 260°C. The XRD of the final decomposed phase is shown corresponding to mostly Cu^0 and some Cu_2O. Cu^0 bulk reference is shown in orange and Cu_2O in green.

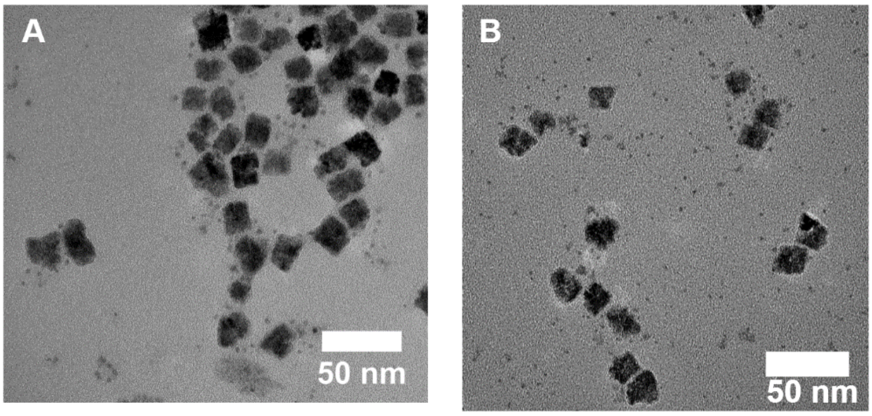

Figure S4. TEM images from the Cu_3N NCs obtained via a reaction with degassing (A) and without degassing (B). Note, these reactions were not performed with the optimized procedure.

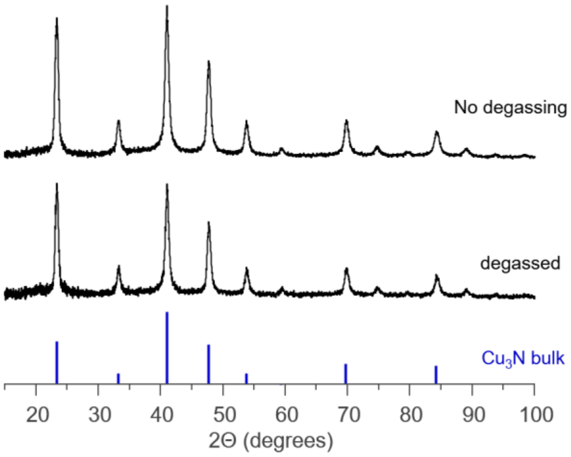

Figure S5. XRD of the Cu_3N NCs obtained via a reaction with degassing and without degassing compared with the bulk.

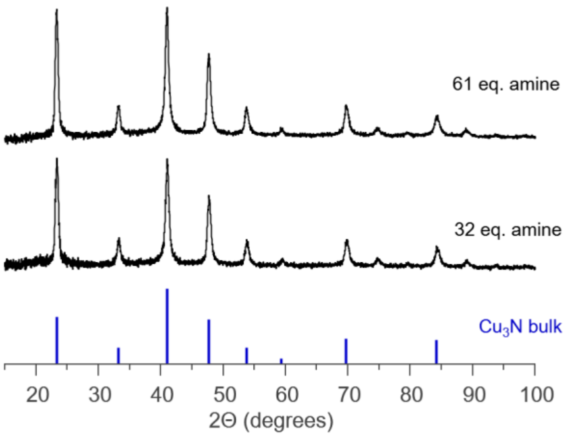

Figure S6. XRD of the Cu_3N NCs obtained with 32 and 61 equivalents of oleylamine compared with the bulk.

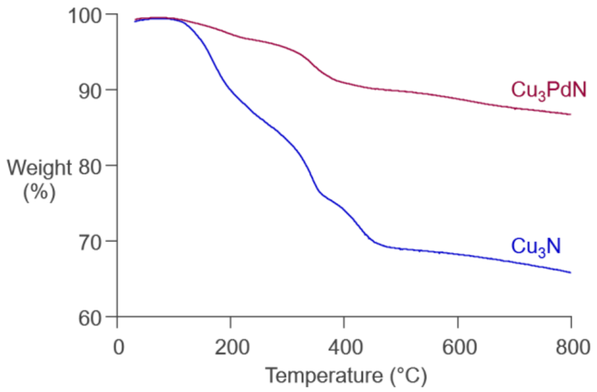

Figure S7. TGA plot of as-synthesized Cu_3N and Cu_3PdN. In the case of Cu_3N, three distinct decompositions are observed from 116°C to 242°C, 242°C to 356°C, and 356°C to 456°C followed by a semi-plateau until 800°C. The end product at 800°C is Cu^0 as judged by the rose gold color and reported literature. In the case of Cu_3PdN, only two decomposition steps can be recognized between 110°C to 276°C and 276°C to 462 °C to finally reach a semi-plateau at 800°C. We believe that the end products are Cu^0 and Pd.

Since Cu_3N is being fully decomposed to Cu^0 at the end of the thermal process, the nitrogen loss should also be taken into account.

$$Cu_3N + organics \xrightarrow{TGA} 3\ Cu$$

We believe that the two first decomposition steps belong to the organics while the last decomposition starting at 356°C belongs to Cu_3N decomposition (bulk Cu_3N decomposes around 400 °C). When starting with Cu_3N + organics, we recover, 65.9 % of the mass as Cu^0 at 800°C. Given the molar mass of copper nitride (204.5 Da) and the molar mass of 3 copper atoms (190.5 Da), the 65.9 % in Cu material corresponds to a theoretical amount of copper nitride as

$$\frac{65.9\ \% \times 204.5}{190.5} = 70.7\ \%$$

29.3 % of the total mass is thus assigned to organics present in the sample. Regarding Cu_3PdN, we assume that at the end of the heating process the end product is Cu^0 and Pd^0 in a 3:1 ratio. The final mass was 86 % percent at 800 °C. Taking into account the molar mass of Cu_3PdN (310.9 Da) and that of 3 copper and 1 palladium atom (296.9 Da), the 86 % in Cu and Pd corresponds to a theoretical amount of Cu_3PdN as

$$\frac{86\ \% \times 310.9}{296.9} = 90\ \%$$

Therefore 10% of our Cu_3PdN sample is organics.

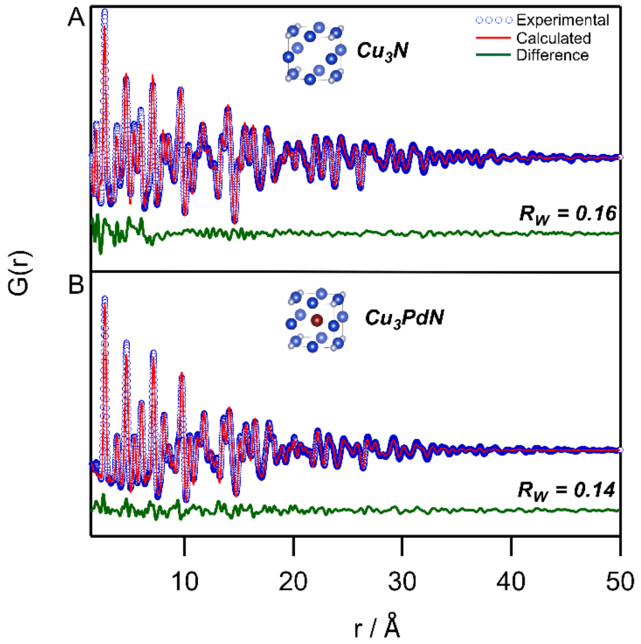

Figure S8. PDF refinement for **(A)** Cu_3N nanocrystals with the cubic (*Pm-3m*) structure. The refined values are given in Table S1. **(B)** Cu_3PdN nanocrystals with Cu_3PdN (*Pm-3m*). The refined values are given in Table S2.

Table S1. Refined values after fitting the Cu_3N nanocrystals with the single-phase cubic (Pm-3m) model.

**Parameter**	**Cu_3N (*Pm-3m*)**
Scale	0.47
a (Å)	3.80
Uiso Cu (Å^2)	0.015
Uiso N (Å^2)	0.027
δ_2 (Å^2)	3.61
Psize (Å)	64.5

Table S2. Refined values after fitting the Cu_3PdN nanocrystals with single-phase Cu_3PdN (Pm-3m) model.

**Parameter**	**Cu_3PdN (*Pm-3m*)**
Scale	0.66
a (Å)	3.83
Uiso Cu (Å^2)	0.015
Uiso N (Å^2)	0.053
Uiso Pd (Å^2)	0.010
δ_2 (Å^2)	4.06
Psize (Å)	50.46

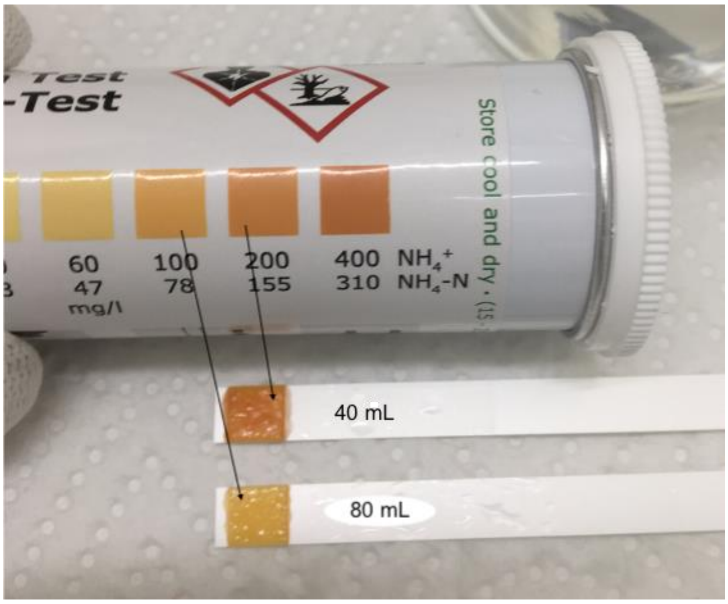

Figure S9. The headspace of the reaction was bubbled into 5 mL of water and then diluted to 40 mL and 80 mL which would result in concentrations of 200 mg/L and 100 mg/L respectively. The color obtained via the test kit corresponds to the expected concentration.

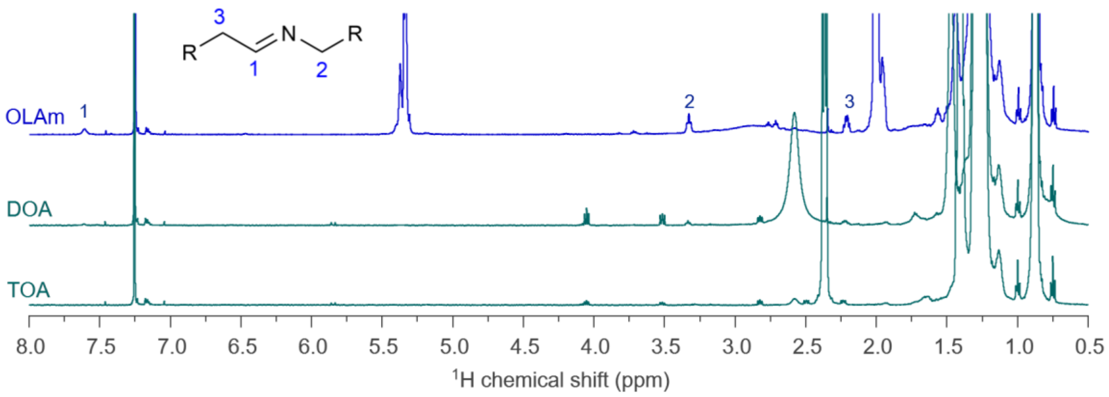

Figure S10. ^{1}H NMR of the crude mixture obtained from the reaction with a primary, secondary, and tertiary amine. Aldimine is mainly detected during the reaction with a primary amine. A little amount of aldimine can be detected with dioctylamine which could be due to direct oxidation of dioctylamine.

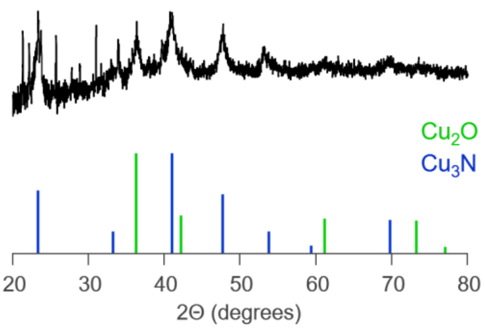

Figure S11. XRD of the NCs obtained after ammonia bubbling into the reaction mixture with $Cu(NO_3)_2$ and dioctylamine. Both Cu_3N and Cu_2O are being formed.

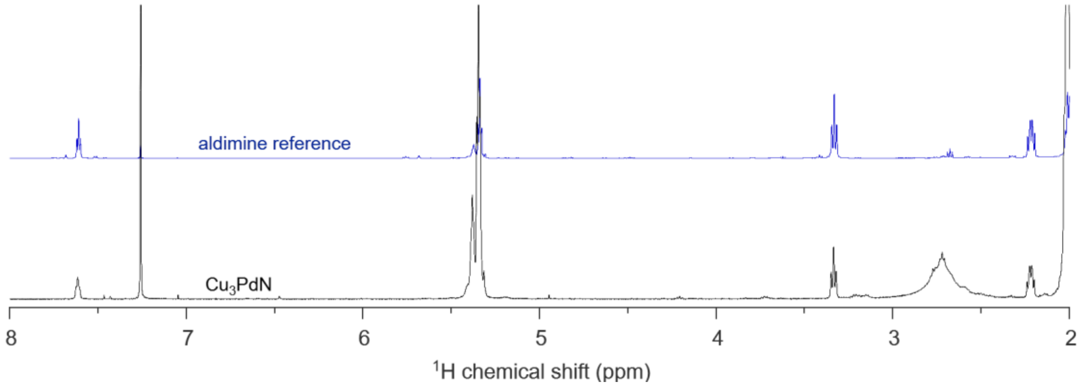

Figure S12. ^{1}H NMR of the crude mixture obtained from the Cu_3PdN reaction after 15 min at 240°C. Aldimine is being formed in the reaction.

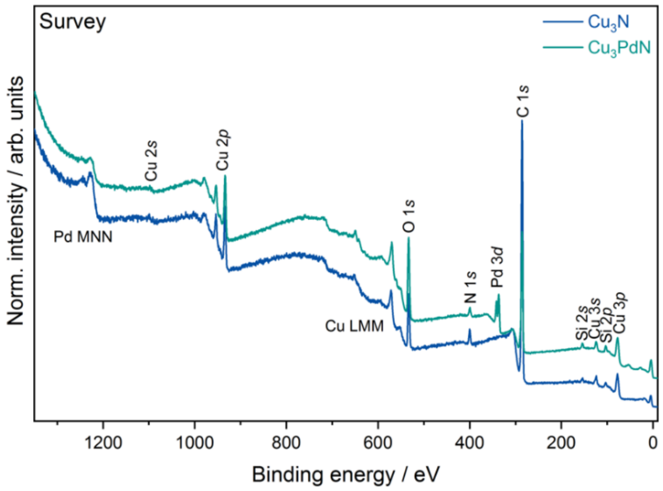

Figure S13. X-ray photoelectron survey spectra of the Cu_3N and Cu_3PdN samples. All major core and Auger lines are indicated.

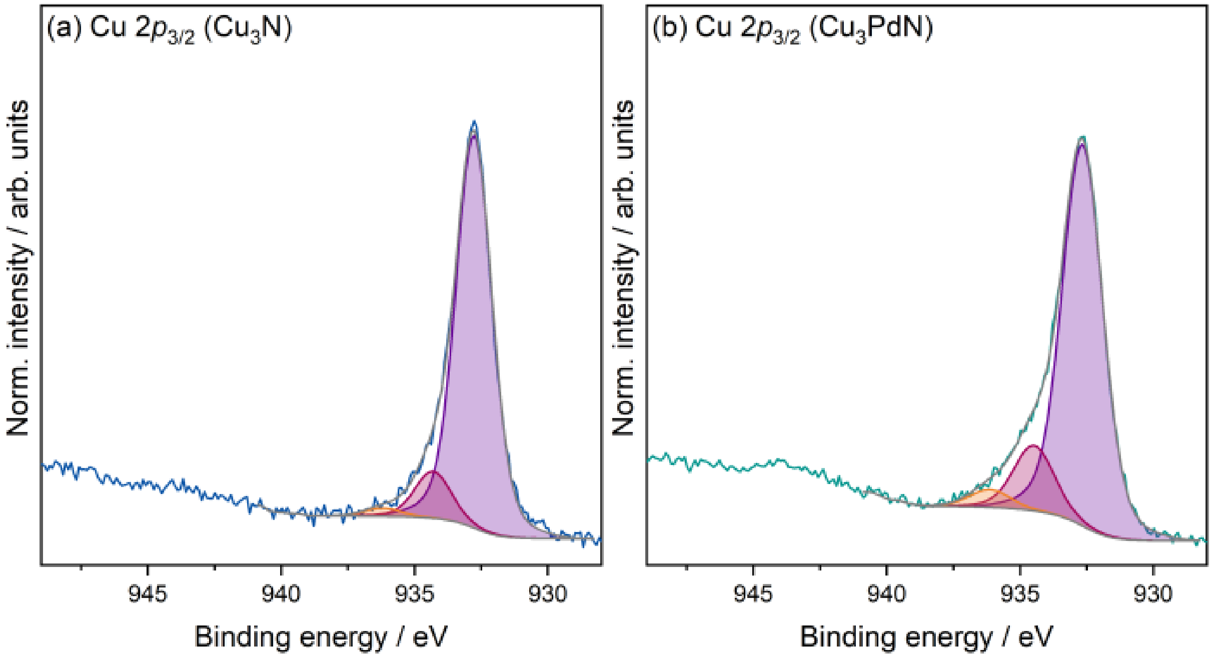

Figure S14. Cu $2p_{3/2}$ X-ray photoelectron core level spectra of (a) the Cu_3N and (b) the Cu_3PdN samples including peak fit analysis.

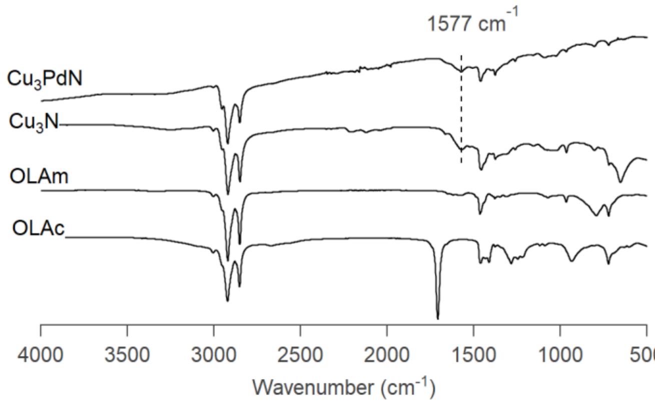

Figure S15. FTIR spectra of Cu_3N NCs after purification compared with oleylamine and oleic acid references. The C=O vibration of bound oleate is detected at 1577 cm^{-1}.

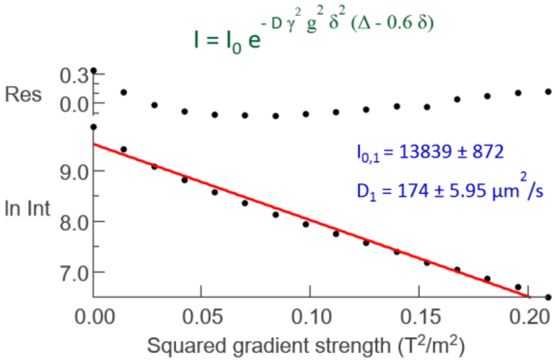

Figure S16. Diffusion decay of the alkene region in the Cu_3N sample in Figure 6A, fitted to the Stejskal-Tanner equation. The average diffusion coefficient corresponds to a solvodynamic size of 4.5 nm (calculated via the Stokes-Einstein equation), which is too small to represent a tightly bound ligand on a 13 nm nanocrystal.

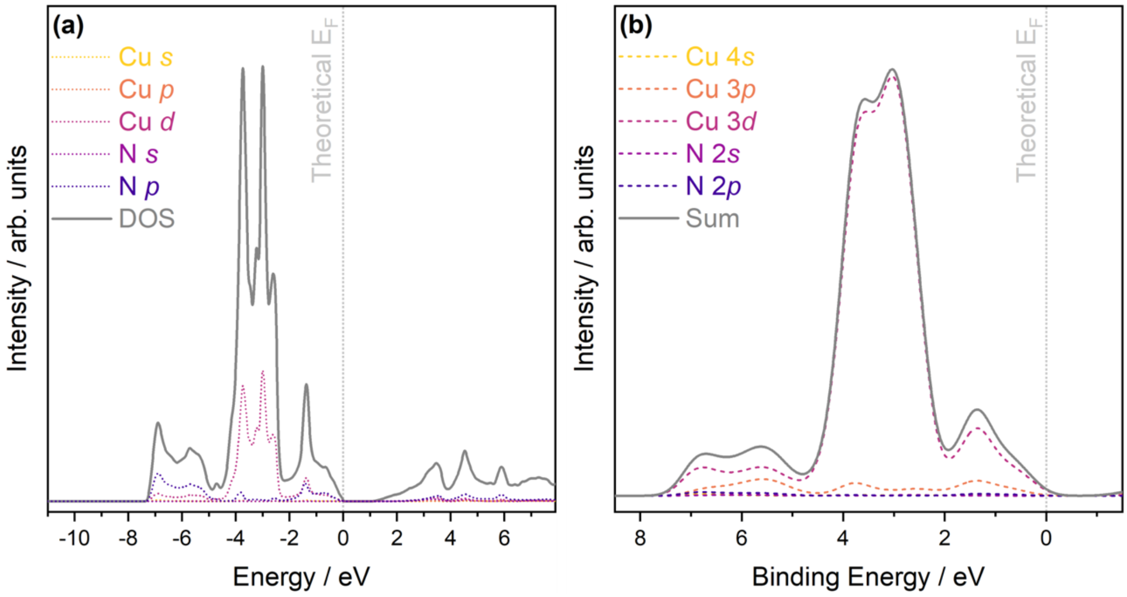

Figure S17. Projected Density of States (PDOS) of Cu_3PdN from density functional theory calculations, including (a) unweighted PDOS with 50 meV Gaussian smearing showing both occupied and unoccupied states, and (b) weighted PDOS with 600 meV Gaussian smearing with the energy axis flipped and only showing the occupied states.

References

[1] D. D. Vaughn Ii, J. Araujo, P. Meduri, J. F. Callejas, M. A. Hickner, R. E. Schaak, *Chem Mater* **2014**, *26*, 6226-6232.

[2] aG. Ashiotis, A. Deschildre, Z. Nawaz, J. P. Wright, D. Karkoulis, F. E. Picca, J. Kieffer, *J Appl Crystallogr* **2015**, *48*, 510-519; bZ. X. Wright CJ, *J Synchrotron Radiat* **2017**, *24(Pt 2)*, 506-508.

[3] aP. Juhas, T. Davis, C. L. Farrow, S. J. L. Billinge, *J Appl Crystallogr* **2013**, *46*, 560-566; bX. Yang, P. Juhas, C. L. Farrow, S. J. Billinge, *arXiv preprint arXiv:1402.3163* **2014**.

[4] aC. L. F. P. Juhás, X. Yang, K. R. Knox and S. J. L. Billinge, *Acta Crystallographica* **2015**, 562-568; bC. L. Farrow, P. Juhas, J. W. Liu, D. Bryndin, E. S. Božin, J. Bloch, T. Proffen, S. J. L. Billinge, *J Phys: Condensed Matter* **2007**, *19*, 335219.

[5] aA. Zakutayev, C. M. Caskey, A. N. Fioretti, D. S. Ginley, J. Vidal, V. Stevanovic, E. Tea, S. Lany, *J Phys Chem Lett* **2014**, *5*, 1117-1125; bS. Lany, *Physical Review B* **2013**, *87*.

[6] V. Stevanović, S. Lany, X. Zhang, A. Zunger, *Physical Review B* **2012**, *85*.

[7] S. Lany, *J Phys Condens Matter* **2015**, *27*, 283203.

[8] aJ. H. Scofield, *Theoretical photoionization cross sections from 1 to 1500 keV.*, **1973**; bA. J Jackson, A. M Ganose, A. Regoutz, R. G. Egdell, D. O Scanlon, *Journal of Open Source Software* **2018**, *3*; cC. F. Kalha, Nathalie; Regoutz, Anna **2020**.

[9] D. Sinnaeve, *Concepts Magn. Reson. Part A* **2012**, *40A*, 39-65.
